# Evaluation of the Antitumor Effectiveness and Toxicity of pH-Sensitive Liposomes Coencapsulating Doxorubicin and Simvastatin in a Murine Breast Cancer Model

**DOI:** 10.1021/acsomega.5c04484

**Published:** 2025-07-08

**Authors:** Jaqueline A. Duarte, Eliza R. Gomes, Geovanni D. Cassali, Pierre Sicard, Sylvain Richard, Philippe Legrand, Andre L.B. de Barros, Elaine A. Leite

**Affiliations:** 1 Department of Pharmaceutical Products, Faculty of Pharmacy, 28114 Federal University of Minas Gerais, Av. Antônio Carlos, 6627, Belo Horizonte 31270-901, Minas Gerais , Brazil; 2 Department of General Pathology, Institute of Biological Sciences, 28114 Federal University of Minas Gerais, Av. Antônio Carlos, 6627, Belo Horizonte, Minas Gerais 31270-901, Brazil; 3 PhyMedExp, IPAM/Biocampus, INSERM/CNRS/Université de Montpellier, Montpellier 34095, France; 4 Institut Charles Gerhardt Montpellier (ICGM), Université de Montpellier, CNRS, ENSCM, Montpellier 34095, France; 5 Department of Clinical and Toxicological Analyses, Faculty of Pharmacy, 28114 Federal University of Minas Gerais, Av. Antônio Carlos, 6627, Belo Horizonte, Minas Gerais 31270-901, Brazil

## Abstract

Combination therapy offers a promising strategy for treating cancer. Research shows that using drug combinations can improve effectiveness against tumors. However, the potential enhancement of adverse effects remains a major concern. The association of statins with anticancer agents has been shown to improve anticancer therapy outcomes and reduce toxicity. This study investigated a pH-sensitive liposomal formulation coencapsulating doxorubicin (DOX) and simvastatin (SIM), referred to as SpHL-D-S, at various molar ratios (DOX:SIM, 1:1, 1:2, and 2:1) for its potential in treating breast tumors. The drug combination at a 1:1 ratio had more significant cytotoxicity than DOX alone on 4T1 breast cancer cell inhibition, with lower IC50 values, and demonstrated a synergistic effect across all concentrations tested. In vivo cardiotoxicity study revealed that 1:1 SpHL-D-S attenuated the short-term cardiotoxic effects of DOX. The antitumor efficacy of the 1:1 ratio, using either the free or encapsulated form, was evaluated in BALB/c mice with 4T1 breast tumors. No significant difference in tumor volume was observed between the SpHL-D-S and DOX:SIM groups after 8 days of treatment. However, the use of SpHL-D-S demonstrated a significant advantage, notably reducing toxicity. Additionally, SpHL-D-S treatment provided important protection for SIM against cardiac and hepatic disorders. In all mice, free DOX promoted cell vacuolization in the heart, which was reduced in animals receiving SpHL-D-S. These findings suggest that the coencapsulation of DOX and SIM in pH-sensitive liposomes may enhance the safety of breast cancer treatment.

## Graphical abstract

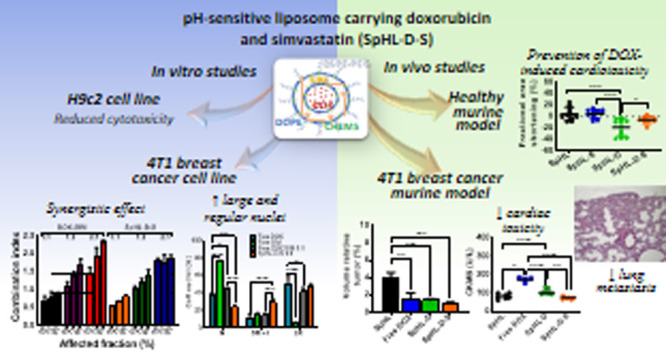

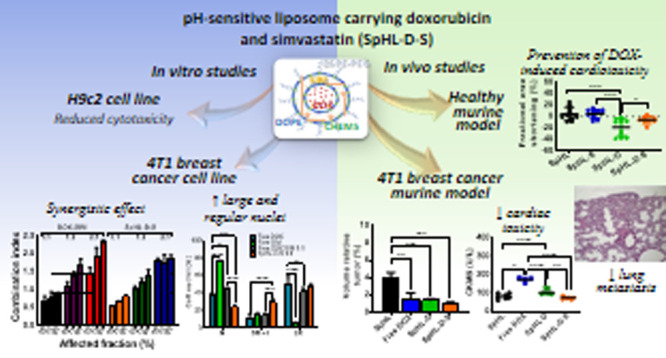

## Introduction

Breast cancer is the leading cause of early mortality among women and represents a significant public health issue worldwide. It is the second most common type of cancer in females, excluding nonmelanoma skin tumors, accounting for 25% of cancers in women worldwide and 29.7% in Brazil.^1^ Among the various subtypes of breast cancer, triple-negative breast cancer (TNBC) tends to have a more aggressive clinical progression. Although TNBC shows higher responsiveness to chemotherapy and despite advancements in treatment options, about 20 to 40% of patients diagnosed with early-stage breast cancer eventually face recurrence or develop metastatic disease.^2^

Doxorubicin (DOX) is the first-line therapy for TNBC. However, its use is limited by two main issues: irreversible cardiotoxicity and low penetration into solid tumors.^3^ Like many other antitumor drugs, DOX is initially effective in killing cancer cells, but resistance often develops over time. To address these challenges in cancer therapy, nanoparticulate systems have emerged as alternative carriers for antineoplastic drugs. These systems can facilitate greater drug accumulation in tumor regions while reducing the overall toxicity. The first liposomal formulation of DOX approved by the Food and Drug Administration for cancer treatment was Doxil. While Doxil has significantly reduced the risk of cardiotoxicity, a clinical study involving patients with advanced breast cancer still reported a cardiac toxicity risk of approximately 11% among those treated with Doxil. As a result, there will continue to be a demand for therapies that offer clinical benefits to patients.^4^

Epidemiological and preclinical studies suggest that combining simvastatin (SIM) with DOX reduces the risk of cardiac tissue damage while maintaining the therapeutic effectiveness of this anthracycline and lowering the incidence of tumor-related deaths.^5^ Additionally, research indicates that SIM may provide beneficial cardiovascular effects through anti-inflammatory and antioxidant mechanisms. These effects could contribute to cardiac protection by minimizing the oxidative stress caused by DOX.^6^ Moreover, studies indicate that combining statins with other antitumor drugs can enhance cancer treatment effectiveness.^7,8^ Although SIM can be administered orally, it undergoes extensive first-pass hepatic metabolism, resulting in less than 5% of the administered dose reaching the systemic circulation. This pharmacokinetic behavior is considered advantageous for the treatment of hypercholesterolemia, where the liver is the primary site of action and systemic exposure is not required. However, in the context of DOX-induced cardiotoxicity and antitumor effects, systemic bioavailability of SIM becomes essential.^9^ A previous study has shown that SIM significantly enhances DOX-induced cytotoxicity, especially when coencapsulated in cubosomes.^10^ It has also been reported that liposomes can maintain a stable ratio of encapsulated drugs for several hours as well as reduce DOX-induced cardiotoxicity.^11,12^ Duarte and collaborators (2023) successfully developed a pH-sensitive liposome containing DOX and SIM (SpHL-D-S) for breast cancer treatment, exploring a potential codelivery strategy. This formulation, termed SpHL-D-S, aims to improve drug delivery to tumor regions by leveraging the unique characteristics of its lipid components, ensuring synchronized distribution of DOX and SIM. *In vitro* studies showed that SpHL-D-S exhibits cytotoxic activity against various subtypes of human breast tumor cell lines, including MCF-7, MDA-MB-231, and SKBR3.^13^ MCF-7 is luminal A (ER^+^/PR^+^/HER2−), hormone-dependent, and less aggressive. MDA-MB-231 is triple-negative (ER–/PR–/HER2−), highly aggressive, with limited treatment options. SKBR3 is HER2-enriched (ER–/PR–/HER2^+^), responsive to HER2-targeted therapies.^14^ Additionally, it demonstrated physicochemical properties, such as size, polydispersity index, zeta potential, encapsulation efficiency, and morphology, which are suitable for *in vivo* applications. A study of releases at different pH levels confirmed the pH-sensitive properties of SpHL-D-S.^13^

The effectiveness of this nanocarrier *in vivo* and its capability to reduce the side effects of DOX require further investigation. Therefore, in this study, we aimed to evaluate the ability of SpHL-D-S to treat a murine TNBC model. The 4T1 breast cancer cell line was selected because it shares substantial molecular characteristics with human TNBC: it is highly tumorigenic, invasive, and capable of spontaneously metastasizing from the primary tumor to distant organs including the lungs, liver, brain, bones, and lymph nodes. Its metastatic behavior closely mirrors that seen in patients. Furthermore, surgical excision of the primary tumor allows the investigation of metastatic progression in a clinically meaningful setting.^15,16^

To the best of our knowledge, this research is the first to evaluate the impact of combining DOX and SIM on 4T1 cancer cells. We initially conducted a study to investigate the synergistic effects of this combination in 4T1 cell lines and to determine the optimal molar ratio for *in vivo* assays. Following this, we examined the formulation’s effect on cardiotoxicity through echocardiographic analysis in healthy mice. Additionally, we assessed the *in vivo* antitumor efficacy and treatment toxicity using the mouse 4T1 model.

## Results and Discussion

### Physicochemical Characterization and Studies on Biological Stability

Initially, formulations with varying molar ratios of DOX to SIM were prepared. In this stage, the concentration of SIM was kept constant while only the DOX concentration was adjusted to achieve the molar ratios of DOX:SIM; i.e., 1:1, 1:2, and 2:1 (Table 1). No significant differences were observed in the physicochemical parameters among the three molar ratios evaluated (Table 1). All samples exhibited average diameters of less than 150 nm and vesicles with a homogeneous size distribution, indicated by a polydispersity index (PDI) of approximately 0.2. The PDI measures the size distribution of the vesicles, and a low PDI reflects a more uniform size distribution, which is desirable for effective drug delivery. Passive targeting of liposomes is known to occur through the enhanced permeability and retention (EPR) effect. This allows nanoparticles to take advantage of the increased presence of fenestrations in the neovasculature to extravasate into tumor sites, leading to higher tumor uptake.^17,18^ Zeta potential values close to the neutral range (−3.0 mV) suggest reduced interaction between the vesicles and plasma proteins after intravenous injection.^19^

**Table 1:** Physicochemical Properties of SpHL-D-S in Different Molar Ratios^a^

	formulations		
	SpHL-D-S (molar ratio)		
parameters	1:1	1:2	2:1
average diameter (nm)	139 ± 3	140 ± 1	136 ± 7
PDI	0.22 ± 0.03	0.19 ± 0.02	0.21 ± 0.04
zeta potential (mV)	–3.4 ± 0.3	–3.6 ± 0.6	–3.7 ± 0.8
DOX concentration (mg/mL)	0.93 ± 0.06	0.50 ± 0.07	1.97 ± 0.11
SIM concentration (mg/mL)	0.72 ± 0.05	0.76 ± 0.04	0.77 ± 0.10

a PDI = polydispersity index; DOX = doxorubicin; SIM = simvastatin; SpHL-D-S = pH-sensitive liposome containing DOX and SIM. Data expressed as average ± standard deviation (*n* = 3).

In terms of encapsulation efficiency, we achieved values close to 100% for DOX and above 60% for SIM. The initial concentration of SIM was set at 1 mg/mL to prepare all formulations containing the coencapsulated drugs in equimolar proportions. Additionally, our previous research demonstrated that the liposome composed of DOX and SIM exhibits suitable physicochemical properties, release behaviors, and pH sensitivity, making it a promising alternative for further breast cancer therapy *in vivo*. The stability of SpHL-D-S, evaluated after reconstituting SpHL-S with a DOX solution, indicated that the concentration of SIM and the encapsulation capacity for DOX remained close to 100% for at least 90 days.^13^

Given the similarity in physicochemical data, the biological stability was assessed by using only SpHL-D-S at a molar ratio of 1:1. *In vitro* biological stability is essential for using liposomes as drug carriers *in vivo*, as they must circulate and retain the drug long enough to effectively access and interact with the target tissue.^20^ The size and PDI of SpHL-D-S 1:1, after incubation in NaCl (0.9% w/v) and RPMI 1640 medium, demonstrated adequate stability under simulated biological conditions (pH 7.4 and 37 °C) for up to 24 h. There was no significant difference in vesicle size compared to that of the control group (Figure 1a,b). In contrast, the measurements of SpHL-S-D after incubation in murine blood plasma showed a diameter that was approximately 1.5-fold smaller than the control, along with a PDI about 1.6 times higher. This property likely results from the presence of two particle populations with different diameters: approximately 90% of the particles measured at 137 nm, while 10% had a diameter of 20 nm, as illustrated in Figure 1c. It is known that at pH 7.0, bovine serum albumin molecules do not aggregate due to electrostatic repulsions, and their size distribution ranges between 5 and 20 nm.^21,22^ This fact supports our findings and enhances the overall robustness of the study. It suggests that the presence of smaller particles in the plasma decreases the average diameter while increasing the PDI. Additionally, the observation of two populations indicates that the plasma proteins did not aggregate within the liposomal vesicle, thereby maintaining its integrity. Consequently, the data demonstrated that SpHL-D-S presented good biological stability regardless of the medium, confirming its suitability for both *in vitro* and *in vivo* tests.

**Figure 1:**
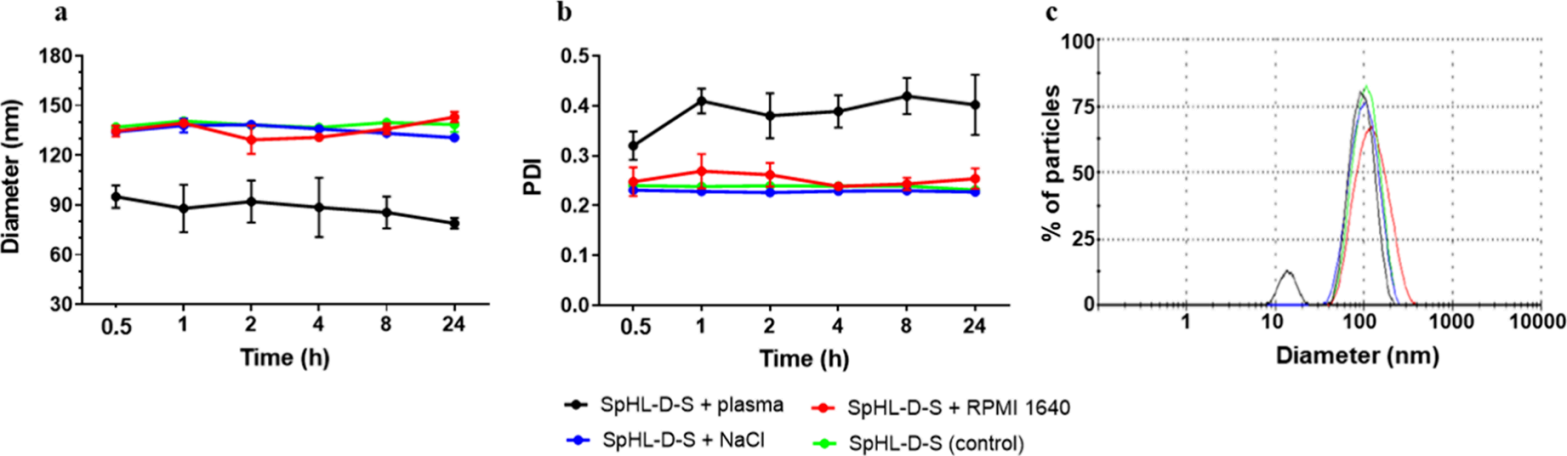
Temporal evolution of average diameter (a) and polydispersity index (b) of SpHL-D-S liposomes upon incubation at 37 °C and pH 7.4 with NaCl (blue), RPMI 1640 medium (red), or murine plasma (black) compared to SpHL-D-S control (without dilution). Data are presented as average ± standard deviation (*n* = 3). (c) Representative dynamic light scattering (DLS) measurements by the intensity of SpHL-D-S. Two peaks with size distributions at 20 and 137 nm were identified for SpHL-D-S incubated with plasma.

### *In Vitro* Studies

Cytotoxicity assessment was performed on murine 4T1 breast cancer tumor cells, which are known to belong to the aggressive and metastatic TNBC subtype.^15,16^ Notably, this study is the first to evaluate cell viability and assess synergism between DOX and SIM against the 4T1 cell line. The cells were incubated with free drugs and SpHL-D-S at various molar ratios, and their viability was analyzed after 48 h of treatment. The results, shown as cell viability percentages and IC50 values, are displayed in Figure 2. No significant differences in viability were found between free DOX and SIM. Evidence indicates that SIM and DOX may exhibit comparable cytotoxic effects in triple-negative breast cancer cells.^23^

**Figure 2:**
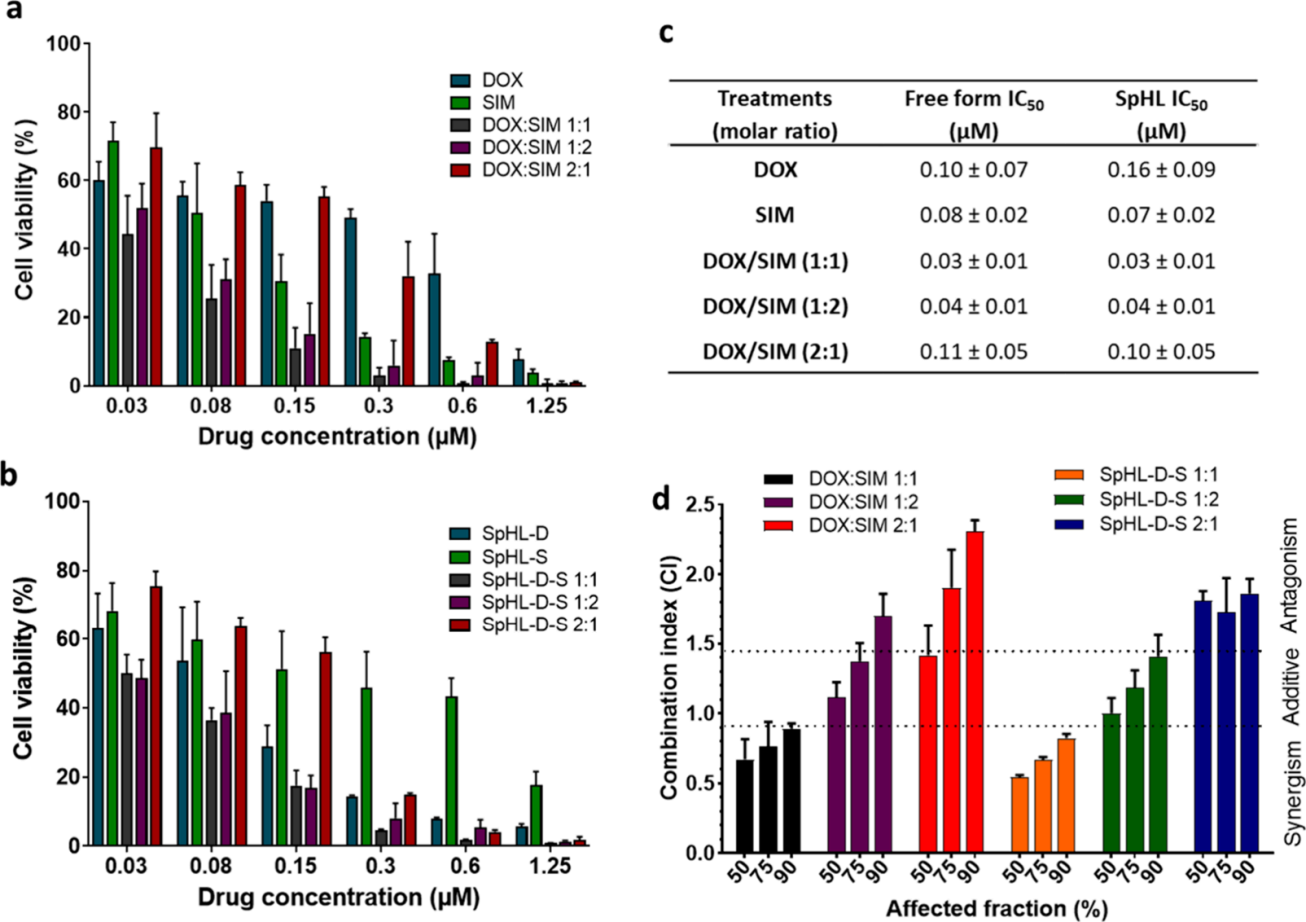
Cytotoxicity of different treatments with (a) free drugs (DOX and SIM) or (b) encapsulated in liposomes against 4T1 murine breast tumor cells. (c) IC_50_ values obtained for cell line 4T1 when exposed to different molar ratios between free DOX and SIM and coencapsulated in SpHL for 48 h. (d) Combination index values of DOX:SIM in free form and coencapsulated in long-circulating and pH-sensitive liposomes versus affected fraction. Three independent experiments were performed on different days and cell passages. Values are expressed as the average ± standard deviation (*n* = 3).

A study by Abdoul-Azize and collaborators reported that both SIM and DOX decreased cell viability in TNBC cells, with SIM exhibiting a more pronounced cellular toxicity profile compared to DOX.^24^ A dose-dependent effect on cell viability was observed with all treatments. At higher concentrations (>0.15 μM), the combination of DOX and SIM at ratios of 1:1 and 1:2 showed significantly greater cytotoxicity than DOX alone (Figure 2a). A similar pattern was observed for SpHL-D-S following higher doses (Figure 2b). However, no significant difference has been detected between the IC50 values for DOX treatment and the DOX:SIM combination, in either free or encapsulated forms (Figure 2c). The IC50 values further confirm that encapsulating the drugs in liposomes did not alter their cytotoxicity against the 4T1 cell line when compared to free treatments. These findings align with previous studies that reported no cytotoxicity when treating cells with blank DSPE-PEG, DOPE, and CHEMS liposomes, indicating that this vehicle is nontoxic.^25,26^

The next step was to evaluate the combined effects of the drugs (synergy, additivity, or antagonism), as the ratio of the drugs can significantly influence these outcomes. An effective combination strategy that can align the pharmacokinetics and biodistribution of drug molecules is highly desirable to maximize their combined effects. By encapsulating drugs within the same nanocarrier, we can ensure that they reach cellular targets simultaneously, enhancing their overall effectiveness. To thoroughly analyze these effects, the data collected from the cytotoxicity study were subjected to median effect analysis by using CalcuSyn software. This analysis focused on varying molar proportions of free and coencapsulated drugs. The combination index (CI) classification for the combinations of DOX and SIM was determined based on different criteria established by Chou.^27^ According to these criteria, a synergistic effect is indicated by a CI of less than 0.9, an additive effect corresponding to a CI between 0.9 and 1.45, and an antagonistic effect indicated by a CI greater than 1.45.^27^

In Figure 2d, we provide the values of the combination indices at cellular inhibition concentrations of 50, 75, and 90%. For anticancer therapies, the ideal scenario is to achieve synergism at all levels of cellular inhibition.^28^ In the case of the 1:1 DOX:SIM combination, a synergistic effect was noted across all inhibition concentrations. For the 1:1 combination, in both free and coencapsulated forms, the mean CIs were 0.80 ± 0.09 and 0.67 ± 0.14, respectively. These results suggest that adding SIM in equimolar amounts to the DOX treatment enhances its effectiveness against 4T1 cells. Therefore, a 1:1 molar ratio is particularly significant for treating lineage 4T1, whether in free form or in a liposomal formulation. It is worth mentioning that although the SpHL-D-S formulation demonstrated a synergistic effect similar to the free drug combination at a 1:1 ratio, pH-sensitive liposomes may offer additional advantages, such as targeted delivery in the tumor region and reduced toxicity.^29^

Nuclear morphometric analyses were performed to evaluate the effects of various treatment forms, including free and coencapsulated drugs, compared to free drugs alone. After exposure to these treatments, cell nuclei were classified into several categories: normal nuclei (N), small and regular (SR) nuclei, which typically correspond to apoptotic cells; irregular (I) nuclei, characteristic of damaged mitotic cells; and large and regular (LR) nuclei associated with senescent cells.^30,31^

Figure 3a displays the morphometric analysis of the nuclear size and irregularity in 4T1 cells. Data revealed that around 37% of the nuclei in cells treated with free DOX had normal morphometry, while around 77% of the nuclei from cells treated with free SIM were also normal. Regarding LR nuclei, around 51% of those in the free DOX group and only 6% in the free SIM group indicated fewer nuclear changes following SIM treatment than DOX. Cotreatment with SpHL-D-S at a molar ratio 1:1 significantly enhanced nuclear enlargement induced by DOX. In this SpHL-D-S 1:1 treatment group, there was a notable decrease (1.5-fold) in the percentage of normal nuclei accompanied by a significant increase (2.7-fold) in LR nuclei when compared to the free DOX treatment. Additionally, a similar pattern was observed when comparing the SpHL-D-S 1:1 treatment with the free DOX:SIM 1:1 combination. These results are consistent with the findings from the synergism test, which demonstrated that the 1:1 molar ratio exhibited synergistic effects.

**Figure 3:**
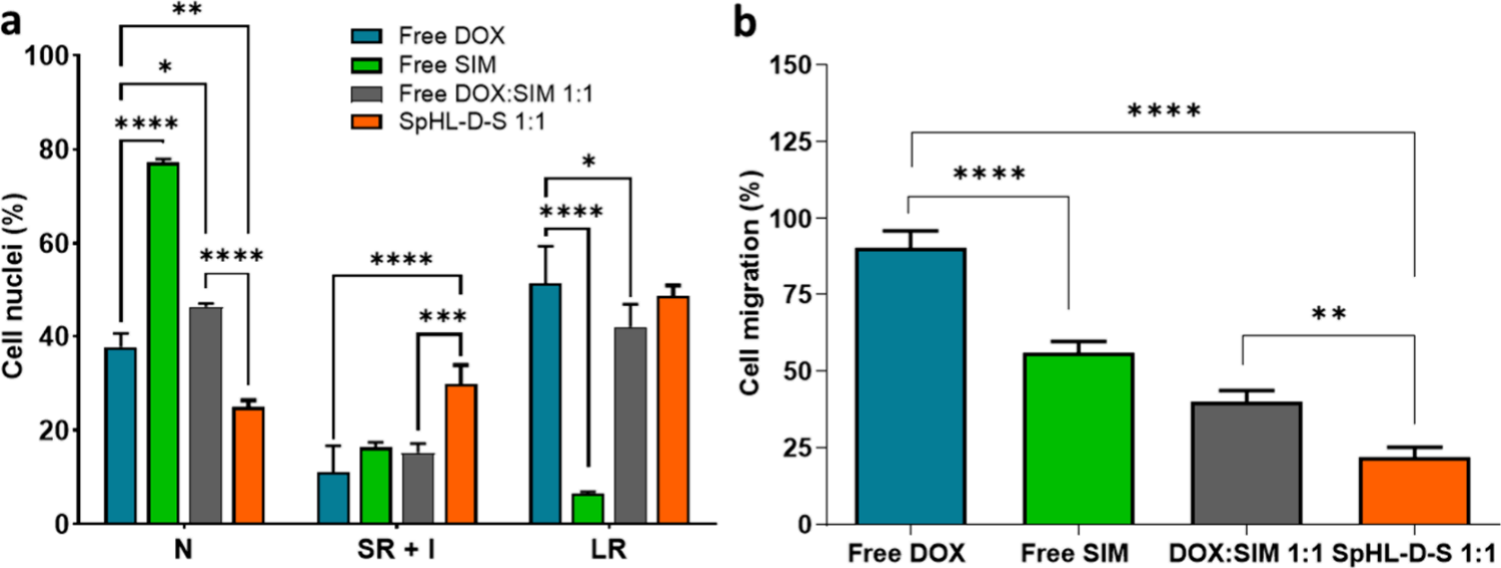
(a) Nuclear morphometric distribution cell nuclei of the 4T1 breast cell line exposed to 80 nM of treatment with free drugs and combined therapy. All groups were pretreated with 80 nM of free drug or combination for 48 h. N is normal; LR is large and regular; SR is small and regular; and I is irregular. (b) Percentage of cell migration of 4T1 cells after exposure to free drug treatment or combined therapy at the total concentration of 80 nM for 24 h. Data are expressed as mean ± standard deviation of three independent experiments. The symbols *, **, ***, and **** represent significant differences for *p* < 0.05, 0.01, 0.001, and 0.0001, respectively (ANOVA followed by Tukey’s multiple comparisons test).

DOX can increase matrix metalloproteinase (MMP) activity, especially MMP-9, which plays a critical role in migrating 4T1 cells.^32^ To evaluate the potential for cell migration, we performed a wound healing test. This test is essential in various stages of the complex metastatic cascade, and its *in vitro* analysis is relevant for developing new prognoses and treatment strategies for cancer. In this experiment, cells were cultured in RPMI 1640 supplemented with 1% fetal bovine serum (FBS) after creating a scratch, ensuring that the observed results were exclusively due to migration. All combined DOX:SIM treatments, whether in the free form or encapsulated liposomes, significantly reduced the percentage of cell migration compared to the free DOX treatment (Figure 3b). Notably, there was a significant difference in migration after treatment with SpHL-D-S 1:1 (22.0 ± 3.2%) compared to free DOX:SIM 1:1 (40.0 ± 3.7%). This promising result indicates that the coencapsulation enhances the efficacy of the combined drugs, presenting a potential breakthrough in inhibiting cell migration in breast cancer cells.

Given the promising results observed with a molar ratio of 1:1, we further examined the selectivity index using the H9c2 cell line. H9c2 is an embryonic rat ventricular myoblast cell line. Although not fully differentiated cardiomyocytes, H9c2 cells retain several cardiac-like features. Due to their sensitivity to oxidative stress and apoptotic stimuli, they serve as a valuable in vitro model for studying the mechanisms of cardiotoxicity, particularly in response to chemotherapeutic agents such as DOX.^33^ The CC50 values for free DOX, SpHL-D, and SpHL-D-S at a 1:1 ratio were 0.15 ± 0.03, 0.26 ± 0.06, and 0.24 ± 0.10 μM, respectively. When we compared these to the IC50 values for 4T1 (Figure 2c), the resulting selectivity indexes were 1.5, 1.6, and 8, respectively. These findings demonstrate the potential of SIM to reduce the risk of cardiac tissue damage caused by DOX while maintaining the therapeutic effectiveness of this anthracycline.^6^

### *In Vivo* Cardiotoxicity Evaluation by Echocardiography Parameters

In order to ensure the *in vivo* safety of SpHL-D-S and allow for the antitumor efficacy investigation, we previously assessed the cardiotoxicity in healthy animals. We evaluated the variation in the trajectory of left cardiac function before and after different treatments. As expected, the SpHL and SpHL-S treatments for 20 days did not significantly alter cardiac function, as measured by ejection fraction, fractional area shortening, left ventricular deformation, or left atrial area (Figure 4a–d). However, SpHL-D-treated mice exhibited a 10–22% reduction in cardiac function and a 50% increase in left atrial volume, clearly demonstrating a moderate but significant cardiotoxic effect of chronic DOX treatment. Interestingly, mice treated with SpHL-D-S did not display major cardiac dysfunction (Figure 4a,b). High-resolution strain analysis and measurements of left atrial dimensions revealed subtle changes, enabling the discrimination between responders and nonresponders to DOX with or without SIM treatment.^34^ These findings highlight the protective potential of SIM in mitigating DOX-induced cardiotoxicity. It has been suggested that SIM exerts cardioprotective effects by attenuating the DOX-induced oxidative stress and mitochondrial dysfunction. It inhibits the mevalonate pathway, reducing Rac1 prenylation and nicotinamide adenine dinucleotide phosphate (NADPH) oxidase-mediated reactive oxygen species (ROS) production. Additionally, SIM activates the PI3K/Akt signaling pathway and upregulates antioxidant enzymes, such as superoxide dismutase and catalase, thereby promoting cardiomyocyte survival.^6,35^ Furthermore, the use of advanced imaging techniques, such as strain analysis, underscores their value in detecting subtle cardiac changes, offering a more refined approach to evaluate treatment efficacy and identify differential responses.

**Figure 4:**
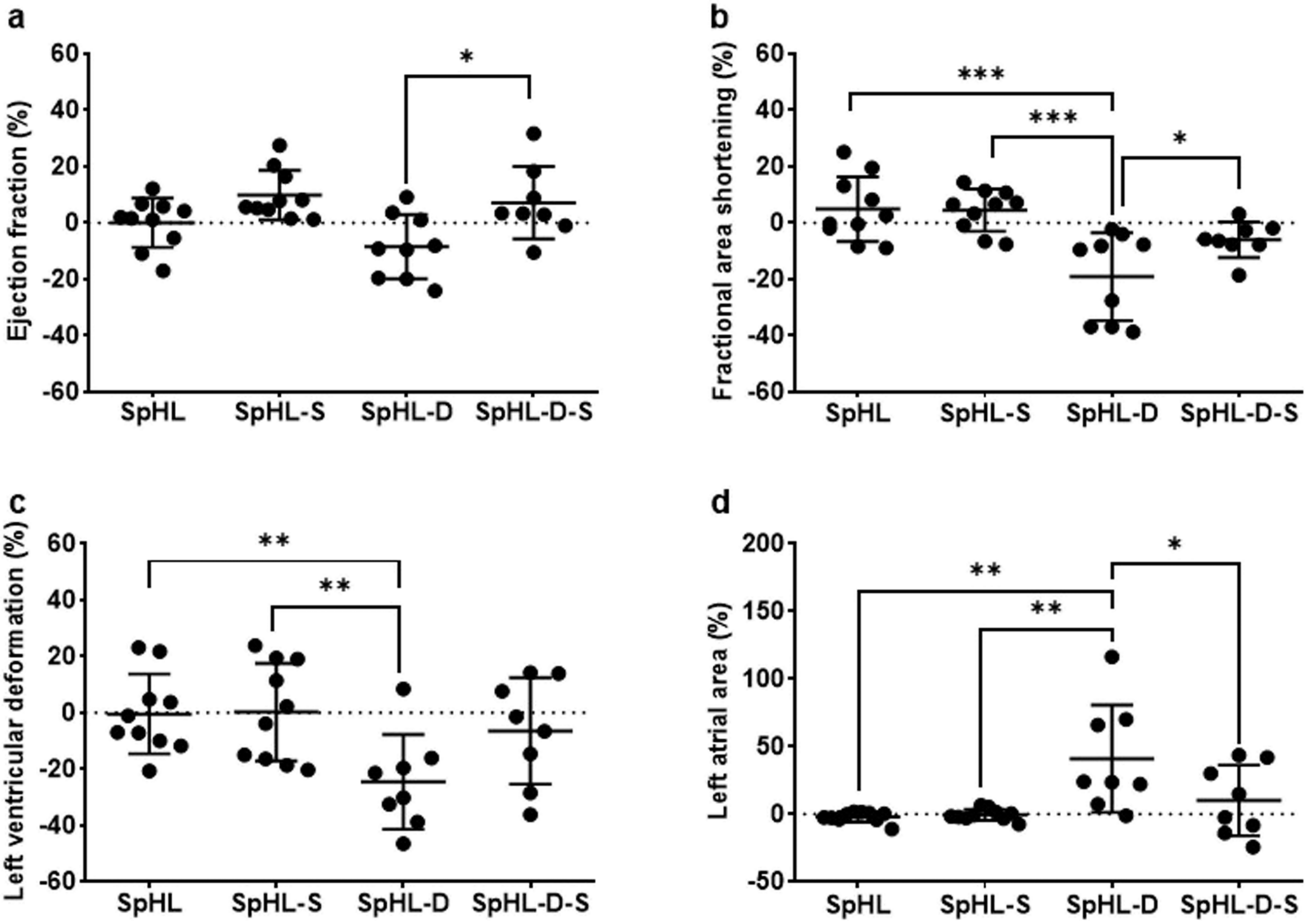
Echocardiography parameter variation: (a) ejection fraction, (b) fractional area shortening, (c) left ventricular deformation, and (d) left atrial area. The parameters were measured in Swiss mice before and after repeated administration of SpHL, SpHL-S, SpHL-D, or SpHL-D-S, and the variation was obtained by ratio after/before × 100%. All data are represented as average ± standard deviation (*n* = 10). The symbols *, **, and *** represent significant differences for *p* < 0.05, 0.01, and 0.001, respectively (two-way ANOVA followed by Tukey’s multiple comparisons test).

### Antitumor Efficacy *In Vivo*

The *in vivo* performance of the different treatments was studied in BALB/c mice with 4T1 tumor, which are known for being highly aggressive and fast-growing, making them a common model in breast cancer research.^16^ The treatments were administered in four doses on days D0, D2, D4, and D6. The control group, which received blank SpHL, underwent a rapid increase in tumor growth, reaching 500 mm^3^ in volume by D8 due to the tumor’s aggressive nature and high-rate cell proliferation. However, all treatment groups exhibited a significant reduction in tumor growth compared to the control group from D4 (Figure 5a).

**Figure 5:**
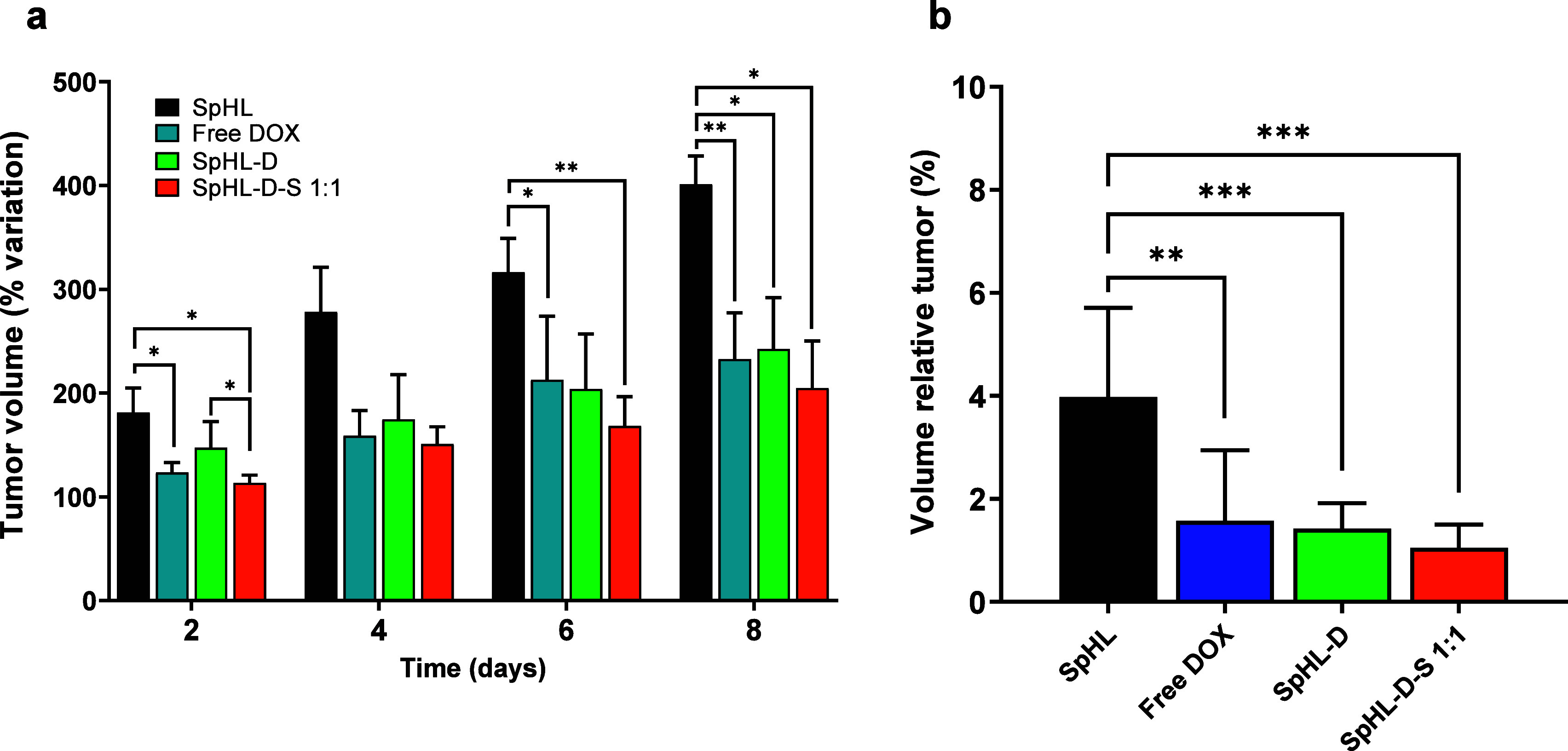
Effect of different treatments on BALB/c mice bearing 4T1 tumor growth. (a) % variation tumor volume. (b) Tumor relative volume (TRV) analysis for each treatment. All data are represented as average ± standard deviation. The symbols *, **, and *** represent significant differences for *p* < 0.05, 0.01, and 0.001, respectively (two-way ANOVA followed by Tukey’s multiple comparisons test).

No significant difference was observed in tumor volume between the animals receiving free DOX and those treated with SpHL-D or SpHL-D-S. However, the group treated with SpHL-D-S showed the lowest tumor growth rate throughout the experiment. This finding is a significant finding and highlights the potential of this formulation. TRV data are presented in Figure 5b. While the antitumor activity of liposomal formulations may be comparable to that of the free forms of the combined drugs, it is important to note that liposomal formulations are often designed to enhance the drug’s safety profile.^36^ Although these liposomal formulations might not significantly change the therapeutic effectiveness, they can help mitigate the systemic toxicity associated with the free forms. Encapsulating the drug in liposomes allows for a more controlled and targeted release, which can reduce adverse side effects and improve the treatment’s tolerability.^37^

### Histopathological Analyses

In all experimental groups, histological analysis was performed on the primary tumor, heart, lungs, liver, and kidneys to identify areas of necrosis and metastasis. This analysis aimed to characterize the primary tumor and potential metastases in other organs. The tumors showed necrosis, regardless of the treatment administered, as depicted in Figure 6. Additionally, the images of the tumors from mice treated with free DOX and SpHL-D revealed invasive cellular infiltration of tumor cells among the muscle fibers (Figure 6b).

**Figure 6:**
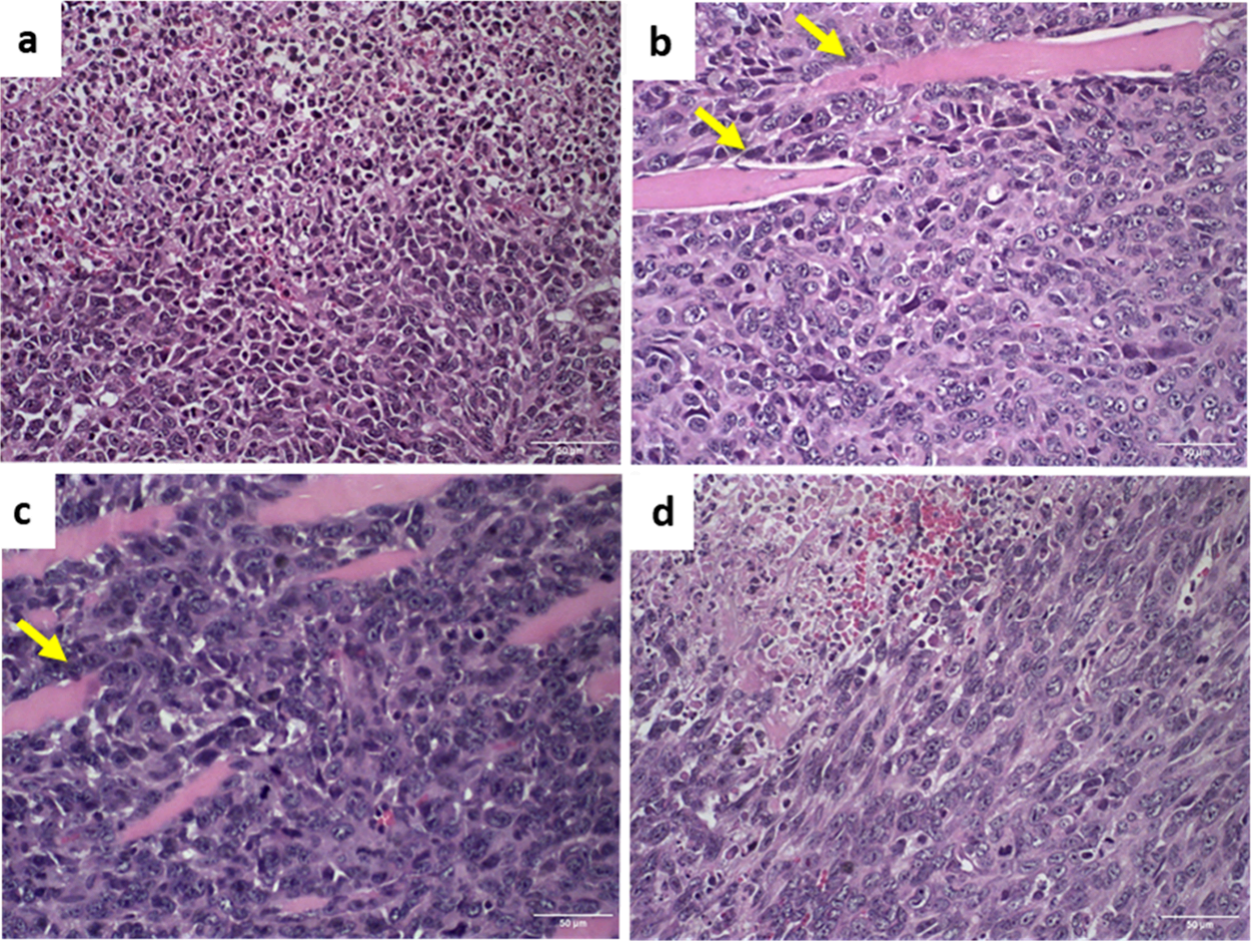
Photomicrographs of tumor tissue from mice in (a) control group, (b) free DOX, (c) SpHL-D, and (d) SpHL-D-S. Yellow arrows indicate the infiltration of tumor cells into muscle fibers of the tissue adjacent to the primary tumor. Hematoxylin–eosin staining, 40× magnification.

Histological analysis of the lungs and liver (Figure 7) showed metastasis foci in mice across all groups except for those receiving free DOX. Mice treated with SpHL-D-S exhibited one or two metastatic foci. In contrast, 25% of the mice treated with free DOX and SpHL-D-S showed no signs of metastatic foci in the liver, while 50% had multiple metastatic foci.

**Figure 7:**
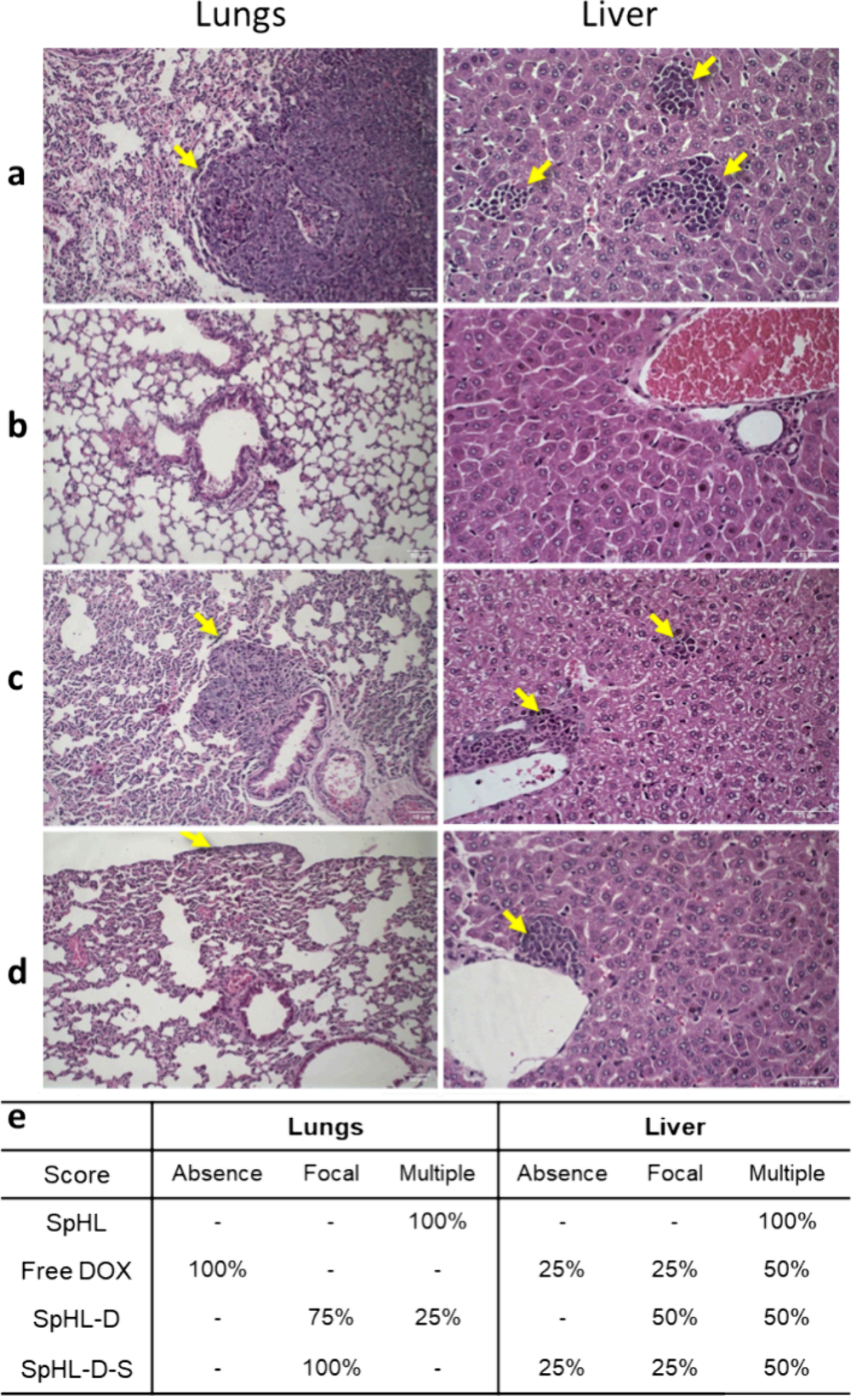
Representative histological sections of lungs and liver from female BALB/c mice carrying a 4T1 breast tumor treated with (a) control group, (b) free DOX, (c) SpHL-D, and (d) SpHL-D-S. (e) Incidence of metastasis in the lungs and liver after intravenous administration of different treatments in BALB/c mice subcutaneously transplanted with 4T1 breast cancer cells. Yellow arrows indicate metastatic foci. Hematoxylin–eosin staining, 20× magnification (lungs), 40× magnification (liver). Absence = no metastasis; focal = 1–3 metastasis foci; multiple = more than 4 metastasis foci.

### Toxicity Assessment after Treatment Regimen in 4T1 Tumor-Bearing Mice

The toxicity of each treatment regimen was assessed by monitoring mortality rates, changes in animal body weight, and biochemical parameters. The treatment with free DOX resulted in a mortality rate of 57% (four out of seven mice). In contrast, mice treated with SpHL-D or SpHL-D-S experienced no deaths, leading to a 100% survival rate. These data suggest that encapsulation markedly reduced the toxicity of the DOX.

The signs of toxicity relating to the body weight of the mice were monitored every 2 days throughout the study. The results are shown in Figure 8a. All treatments containing DOX-induced significant weight loss compared with the control group. Furthermore, the animals that received SpHL-D-S experienced significantly less loss of weight than those treated with free DOX.

**Figure 8:**
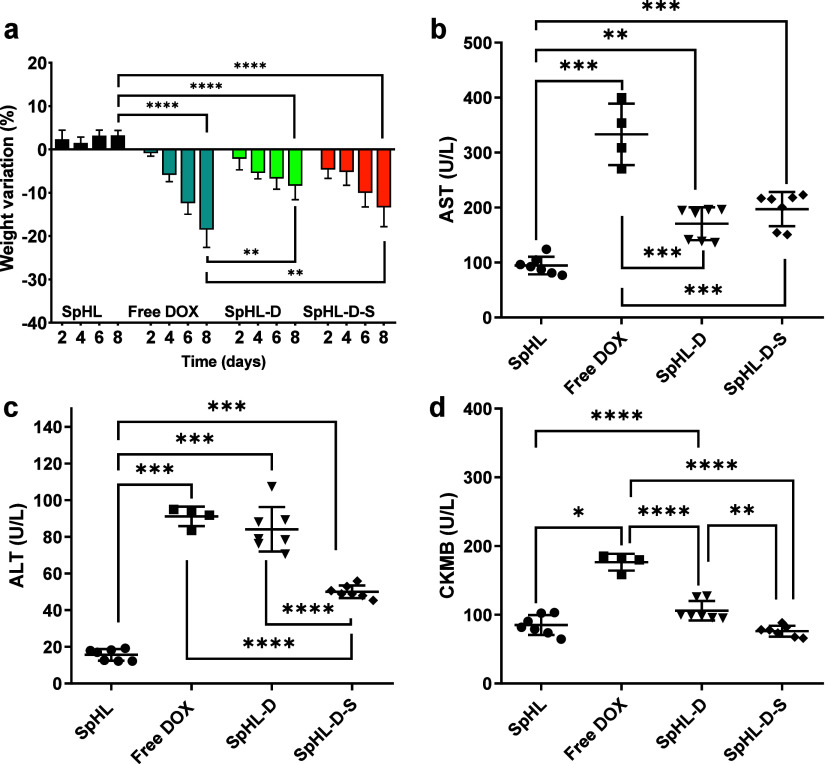
(a) Percentage of variation in body weight of BALB/c mice subcutaneously transplanted with 4T1 breast cancer cells after different treatments for 8 days. Biochemical parameters (b) AST levels, (c) ALT levels, and (d) CK-MB levels evaluated in BALB/c mice bearing 4T1 tumor after different treatments. The symbols *, **, ***, and **** represent significant differences for *p* < 0.05, 0.01, 0.001, and 0.0001, respectively (two-way ANOVA followed by Tukey’s multiple comparisons test). Data are expressed as the mean ± standard deviation of the mean (*n* = 7).

A biochemical analysis was performed to assess hepatic, renal, and cardiac toxicity. In terms of liver toxicity, reports are showing that DOX increases serum levels of alanine aminotransferase (ALT) and aspartate aminotransferase (AST) in mice.^38^ This liver damage is attributed to the formation of free radicals and the generation of reactive oxygen species, which promote oxidative damage to cells. These changes can lead to apoptosis or necrosis of hepatocytes, leading to a significant rise in liver enzymes in the blood, particularly ALT and AST.^39^ As illustrated in Figure 8b, AST enzyme activity was higher in all treatment groups compared to that in the control group. While SIM is usually considered safe, some clinical trials have reported AST elevations of up to three times the upper limit of normal.^40,41^ Statins are associated with elevated AST values. However, these abnormalities are typically clinically insignificant, with rare instances of liver damage that are generally reversible without intervention.^42^ Notably, animals treated with SpHL-D-S produced AST values approximately 1.8 times lower than those treated with free DOX, suggesting a potential reduction in hepatic toxicity.

ALT levels were significantly elevated in all groups treated with DOX (Figure 8c). However, the SpHL-D-S group exhibited notably lower ALT levels, approximately 1.9 times lower than those of the free DOX group. These results suggest that SpHL-D-S effectively reduces the hepatotoxicity associated with DOX treatment.

An increase in serum creatine kinase isoform MB (CK-MB) levels indicates cardiac injury. DOX generates ROS, which triggers cardiomyocytes to release biomarkers associated with heart failure. In addition, the existing literature supports the hypothesis that statins, such as SIM, may alleviate DOX-induced oxidative stress through various antioxidant effects. The mechanisms include reducing NADPH oxidase activity, suppressing the uncoupling of endothelial nitric oxide synthase, and inhibiting DNA damage caused by hydrogen peroxide.^6,35,43,44^ Here, we found a significant increase in CK-MB levels (1.9 times higher) in animals treated with free DOX and the DOX:SIM combination compared to the control group (Figure 8d). Specifically, CK-MB levels in the free DOX and DOX:SIM groups were 1.9 times and 1.5 times higher, respectively, than those of the control group. In contrast, the CK-MB values in animals treated with SpHL-D-S were significantly lower (approximately 1.5 times less) when compared to the free DOX and DOX:SIM groups.

These findings align with the histological analysis of cardiac tissue, suggesting that SIM provides cardioprotective effects against the adverse effects of DOX. Notably, animals in the control group presented cardiac tissue with a typical architecture. In contrast, the group treated with free DOX exhibited large areas of vacuolization of cardiomyocytes, whereas the group receiving the drug combination exhibited either minimal or no vacuolization (Figure 9). The presence of vacuoles within myocardial fibers may indicate deleterious effects induced by DOX.^45^ This reduction in cardiomyocyte vacuolation may reflect the beneficial pleiotropic cardiovascular effects of SIM, which are attributed to its anti-inflammatory and antioxidant properties that can alleviate oxidative stress caused by DOX.^46^ Additionally, a clinical cohort study reported a significantly lower risk of heart failure in breast cancer patients who received SIM during DOX chemotherapy, further supporting the potential cardioprotective role of SIM.^47^

**Figure 9:**
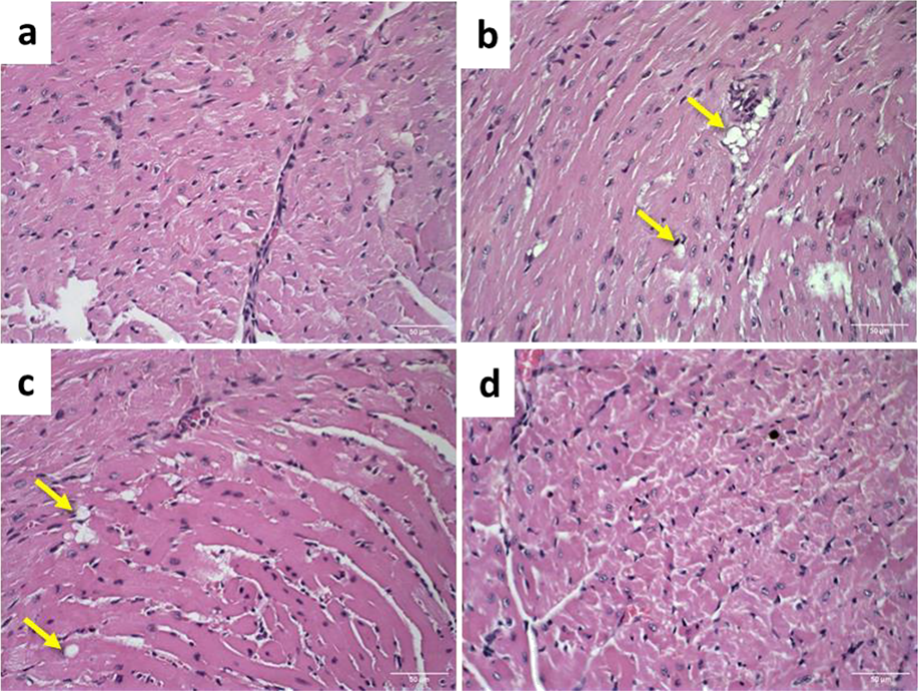
Histological sections of hearts from female BALB/c mice carrying a 4T1 breast tumor treated with (a) the control group, (b) free DOX, (c) SpHL-D, and (d) SpHL-D-S. Yellow arrows indicate vacuolation of cardiomyocytes. Hematoxylin-eosin staining, 40× magnification.

In the analysis of renal toxicity parameters, specifically urea and creatinine levels, we observed no significant differences in any of the treated groups compared with the control group. These data are consistent with the renal histopathology results, since all groups presented animals with preserved tissues and typical anatomical architecture (data not shown).

## 4 Conclusions

*In vitro*, 1:1 SpHL-D-S demonstrated synergistic effects, indicating that it has more significant cytotoxicity than free DOX. *In vivo*, the SpHL-D-S formulation exhibited lower toxicity than free DOX in a mouse experimental model and a significant protective effect of SIM against cardiac and hepatic disorders. More specifically, chronic DOX treatment induces moderate cardiotoxicity, as evidenced by reduced cardiac function and increased left atrial volume. SIM cotreatment effectively mitigates these effects, with high-resolution imaging revealing subtle cardiac changes that help distinguish treatment responses. These findings highlight the potential of SIM as a cardioprotective agent and underscore the importance of advanced imaging techniques for detailed cardiac assessment. Altogether, our data indicate that coencapsulation of DOX and SIM into pH-sensitive liposomes might be an important strategy to improve the safety and efficacy of breast cancer treatments.

## Materials and Methods

### Materials

1,2-Dioleoyl-*sn*-glycero-3-phosphoethanolamine (DOPE) and 1,2-diestearoyl-*sn*-glycero-3-phosphoethanolamine-*N*-[amino­(polyethylene glycol)-2000 (DSPE-PEG2000) were supplied by Lipoid GmbH (Ludwigshafen, Germany). Cholesteryl hemisuccinate (CHEMS) and doxorubicin hydrochloride (DOX) were purchased from ACIC Chemicals (Brantford, ON, Canada). Phosphate-buffered saline (PBS), sodium hydroxide, 4-(2-hydroxyethyl)­piperazine-1-ethanesulfonic acid (HEPES), ammonium sulfate, and sodium bicarbonate were obtained from Sigma-Aldrich (St. Louis, USA). Polysorbate 80 (Tween 80) was provided by Croda Inc. (Edison, USA). Sodium chloride and HPLC-grade methanol were purchased from Merck (Frankfurt, Germany). SIM was purchased from Fagron (São Paulo, Brazil) with a purity greater than 98.0%. The other reagents used were of analytical grade.

4T1 murine breast cancer cells were obtained from the American Type Culture Collection (ATCC) (Manassas, USA). Roswell Park Memorial Institute (RPMI) 1640 medium, penicillin, streptomycin, and fetal bovine serum (FBS) were obtained from Gibco Life Technologies (Carlsbad, USA). Sulforhodamine B (SRB), tris­(hydroxymethyl)­aminomethane (Tris base), and trypsin were obtained from Sigma-Aldrich (St. Louis, USA). The water used in the experiments was purified using a Milli-Q distillation and deionization equipment (Millipore, MA, USA). Hoechst 33258 (Thermo Fisher Scientific—Waltham, MA, USA).

### Preparation and Characterization of Liposomal Formulations

pH-sensitive liposomes containing DOX and SIM (SpHL-D-S) were prepared using the lipid film hydration technique as previously described.^48^ Briefly, DOPE, CHEMS, and DSPE-PEG2000 (in a molar ratio of 5.7:3.8:0.5, respectively) were dissolved in chloroform to a total lipid concentration of 10 mmol L^–1^. A chloroform solution of SIM (1 mg/mL) was added to a round-bottom flask, which was subjected to evaporation under reduced pressure in a water bath at 30 °C and rotation of 150 rpm until a thin lipid film was obtained. NaOH solution, sufficient to promote complete ionization of the CHEMS, was added to the lipid film and then hydrated with an ammonium sulfate solution (pH 7.4). Calibration of liposomal vesicles was performed using ultrasound (model CPX 500; 500 W, Cole-Parmer Instruments, Vernon Hills, Illinois, USA) and a Stepped microtip S&M 630-0418 rod, with 21% amplitude, for 5 min. This preparation was then subjected to ultracentrifugation at 50,000 rpm at 4 °C for 2 h (Beckman Coulter Optima 32L-80 XP ultracentrifuge, USA) to eliminate external ammonium sulfate. The SIM concentration was determined by high-performance liquid chromatography (HPLC) analysis. After that, DOX was remotely loaded by a transmembrane gradient of ammonium sulfate to obtain the desired molar ratio. Liposomes containing only SIM (SpHL-S) were prepared as described above without the addition of DOX, and liposomes containing only 1 mg/mL DOX (SpHL-D) were prepared without adding SIM during film formation. Blank liposomes (SpHL) were prepared without adding SIM and DOX.

The mean diameter and polydispersity index (PDI) of the formulations were measured by dynamic light scattering (DLS). The zeta potential value was determined by DLS combined with the electrophoretic mobility. The DOX and SIM content was measured by HPLC as described by Duarte et al.^13^

### *In Vitro* Studies

#### Stability in Different Media

The stability of SpHL-D-S was investigated in the presence of different fluids, selected to simulate the behavior of vesicles in biological assays (*in vitro* and *in vivo*). SpHL-D-S was diluted four times in NaCl (0.9% w/v), RPMI 1640 culture medium supplemented with 10% (v/v) FBS, and murine plasma. SpHL-D-S undiluted was also evaluated as a study control.

The suspensions were incubated at 37 °C under agitation at 150 rpm for 24h. Aliquots were collected at predetermined times (0.5, 1, 2, 8, and 24 h) to measure size and PDI, as previously described.

#### Cytotoxicity Assay and Synergism Analysis

The murine cancer cell line 4T1 was cultured in RPMI 1640 supplemented with 10% FBS in the presence of penicillin (100 U/mL) and streptomycin (100 μg/mL) and maintained at 37 °C and 5% CO_2_ in a humidified atmosphere. Before the experiments, the cell lineage was screened for mycoplasma by polymerase chain reaction (PCR), with negative results.

H9c2 cardiomyocytes were cultured in Dulbecco’s modified eagle medium (DMEM) supplemented with 10% FBS and 1% penicillin–streptomycin solution.

Cell viability was assessed using the sulforhodamine B (SRB) assay. Briefly, 4T1 cells were seeded at 5 × 10^3^ cells per well of 96-well plates and incubated at 37 °C and 5% CO_2_. After 24 h postseeding, solutions of free DOX, free SIM, and their combinations (DOX:SIM) in molar ratios of 1:1, 1:2, and 2:1, respectively, and liposomal forms at the same combination ratios were added to each well (DOX concentration ranged from 5 to 0.005 μM). After 48 h of treatment, 100 μL of 10% trichloroacetic acid (TCA) was added to each well and incubated for 1 h at 4 °C, and then the plates were washed with water. 100 μL of sulforodamine B was added and then incubated for 30 min. Finally, the wells were washed with 1% acetic acid (v/v), and protein-bound dye was solubilized with 100 μL of a 10 mM Tris base solution (pH 10.5). Absorbance was determined at 510 nm using a SpectraMax Plus 384 spectrophotometer (Molecular Devices, Sunnyvale, USA). IC50 (50% inhibitory concentration of 4T1 cell growth) values were determined by using GraphPad Prism 6.0 (GraphPad Software, La Jolla, California, USA). By analyzing the combination index (CI), calculated using CalcuSyn software (Biosoft, Ferguson, Missouri, USA), the effect of the combinations (free or encapsulated in liposomes) in terms of synergism, additive, or antagonism was determined. Thus, a CI value >0.9 indicates synergism (greater effect than expected from individual agents), a CI between 0.9 and 1.45 indicates additive effect (expected combined effect), and a CI > 1.45 indicates antagonism (less effect than expected).^27^

Cytotoxicity of the formulations was also assessed in the H9c2 cardiomyocyte cell line. Cells were seeded in a 96-well plate (1 × 10^5^ cells per well) and incubated at 37 °C and 5% CO_2_. After 24 h postseeding, cells were treated with free DOX, SpHL-D, and SpHL-D-S (DOX concentration varying from 5 to 0.005 μM) for 48 h. After treatment, the SRB assay protocol described above was performed. CC50 (the concentration that causes 50% inhibition of H9c2 proliferation) values were determined using GraphPad Prism 6.0 (GraphPad Software, La Jolla, California, USA) and the selectivity index was calculated by the ratio CC50/IC50.

#### Nuclear Morphometric Analyses

4T1 murine breast carcinoma cells were plated at a 2.0 × 10^5^ cells/well density in 6-well plates and incubated at 37 °C for 24 h. After the incubation time, the cells were treated with DOX, SIM, and mixtures of free or encapsulated DOX:SIM at molar ratios of 1:1, 1:2, and 2:1 at a concentration of 80 nM and incubated for 48 h. Hereafter, the cells were fixed with 4% v/v formaldehyde phosphate-buffered saline (PBS) for 10 min and then stained with Hoescht 33342 solution (0.2 μg/mL) at room temperature for 10 min. The nuclei were classified according to size and shape in fluorescence images captured by using an AxioVert 25 microscope with a Fluo HBO 50 fluorescence module connected to the Axiocam MRC camera (Zeiss, Oberkochen, Germany). Analysis was carried out with 300 nuclei per treatment using ImageJ 1.50i, and the classification adopted was normal (N), small and regular (SR), large and regular (LR), and irregular (I). SR nuclei typically correspond to apoptotic cells, while LR and I correspond to senescent cell nuclei.^30^

#### Migration Test

To study two-dimensional migration, 4T1 cells were plated at 2.0 × 10^5^ cells per well density in 12-well plates. These cells were then incubated at 37 °C for 24 h in RPMI 1640 medium with 1% FBS. The confluent cell monolayer was ″wounded” by scraping off into individual wells with a 10 μL pipet tip. At that time, images in phase contrast were captured using an AxioVert 25 microscope with an Axiocam MRC camera attached (Zeiss, Oberkochen, Germany) and considered as a reference (″zero wound″). Subsequently, 1 mL of medium (RPMI 1640 with 1% FBS) containing the different treatments (DOX, SIM, and mixtures of DOX:SIM free or encapsulated in molar ratios 1:1; 1:2, and 2:1) was added to each well at a concentration of 80 nM. The plates were incubated at 37 °C for 24 h, and then cells were fixed with 4% v/v formaldehyde in PBS for 10 min. Images along the “treated wounds” were obtained with phase contrast. Wound areas were obtained using the MRI Wound Healing Tool plugin for the free version of ImageJ 1.45 software (National Institutes of Health, Bethesda, USA).

### *In Vivo* Studies

#### Cardiotoxicity Study

Cardiac function was assessed in anesthetized animals using the Vevo 3100 high-frequency ultrasound system (FUJIFILM VisualSonics) equipped with a 40 MHz center frequency MX550D linear array probe, as previously described.^49^ Healthy Swiss mice (*n* = 10 mice per group) were treated with blank SpHL, SpHL-D, SpHL-S, or SpHL-D-S through the tail vein with repeated doses equivalent to 4 mg/kg/day of DOX every other day in a total of five administrations, reaching a cumulative dose of 25 mg/kg. The ventral thorax hair was removed using a depilatory cream, and the mice were secured in a supine position on the imaging stage. Vital signs were continuously monitored throughout the imaging process. To evaluate cardiac function and geometry, we acquired short-axis (SAX) B-mode and M-mode SAX images of the left ventricle. Two-dimensional image analysis was performed using VevoLab software (v3.9.0, FUJIFILM VisualSonics), and the cardiac strain was assessed using VevoStrain software (v1.0, FUJIFILM VisualSonics). Offline image analyses were performed using dedicated Visual Sonics Vevo 3100 version 3.1.0 software.

#### Antitumor Efficacy

Female BALB/c mice, aged 6 to 8 weeks old and weighing between 18 and 22 g, were obtained from the Bioterism Center of UFMG (CEBIO/UFMG). The mice were unrestricted to food and water and housed in ventilated racks with controlled temperature and humidity, following a 12 h light and 12 h dark cycle. All studies were approved by the Ethics in Animal Use Committee of UFMG (CEUA/UFMG) under protocol number 190/2021.

A suspension of 4T1 cells (1 × 10^6^ cells/mL) was prepared in PBS to establish a xenographic breast tumor. Aliquots of 100 μL of this suspension were injected subcutaneously into the right flank of BALB/c mice with an insulin syringe and a 13 mm × 0.33 mm needle. Seven days later, when the tumor volume reached approximately 100 mm^3^, the animals were randomly divided into seven groups (*n* = 7 for each group) and received free DOX or SpHL-D (5 mg/kg) and SpHL-D-S (5 mg/kg DOX and 3.85 mg/kg of SIM). The mice received four administrations every other day through the tail vein, using an insulin syringe fitted with a 13 mm × 0.33 mm needle, with each injection not exceeding a volume of 200 μL. Free drug solutions were prepared immediately before injection. DOX was dissolved in a 0.9% (w/v) NaCl solution at 2 mg/mL. The molar ratio chosen was 1:1 DOX:SIM due to the best *in vitro* results for this murine breast cancer strain.

Throughout the study, tumors were measured with a caliper (Mitutoyo, MIP/E-103) every other day from the first day of treatment (D0) until 2 days after the last administration (D8). Tumor volume (*V*) was calculated from the following equation, where *d*1 and *d*2 are the smallest and largest diameters, respectively:^50^

$$V={\left(d1\right)}^{2}\times d2\times 0.5$$ 1

Tumor relative volume (TRV) was calculated using the following equation:

TRV=(VinD8)/(VinD0) 2

On D8, mice were anesthetized using ketamine and xylazine (80 and 10 mg/kg, respectively) and then euthanized by exsanguination. Blood collected by puncture of the brachial plexus in tubes containing 10% w/v EDTA solution was used to evaluate cardiac, renal, and hepatic biochemical parameters.

In addition, primary tumor, lung, heart, kidney, and liver were collected for histopathological analysis. These organs were stored in 10% (v/v) buffered formalin for 24 h. Then, the samples were dehydrated in alcohol and included in paraffin blocks, sectioned with a thickness of 5 μm, placed on glass slides, and stained with hematoxylin–eosin (HE). The slides were evaluated by a trained pathologist, and the images were captured by a camera connected to an Olympus BX-40 optical microscope (Olympus, Tokyo, Japan). The number of metastasis foci in the lungs and liver was counted in individual animals and followed semiquantitative score: 0, no metastases detected; 1–3 metastatic foci; multiple foci (>4 metastatic foci).

#### Toxicity Evaluation after Treatment Regimen in 4T1 Tumor-Bearing Mice

The toxicity of different treatments was observed by evaluating changes in the body weight, mortality, and biochemical parameters. The mice’s body weight was monitored every 2 days along with the treatment until euthanasia. Weight changes were expressed as the percentage changes in the initial body weight.

For the biochemical analyses, on the last day of the study, the mice were anesthetized with a mixture of ketamine (80 mg/kg) and xylazine (10 mg/kg), and blood was collected by puncture of the brachial plexus in tubes containing anticoagulant (0.1% w/v EDTA). The collected whole blood was spun at 3000 rpm for 15 min. The plasma obtained was used to quantify the liver, kidney, and heart parameters. Alanine aminotransferase (ALT) and aspartate aminotransferase (AST) measurements were performed to determine liver function. Nephrotoxicity was determined by measuring the concentrations of urea and creatinine. Cardiac function was assessed by measuring creatine kinase isoform MB (CK-MB). All biochemical tests were performed through spectrophotometric analysis in a semiautomatic analyzer model Bioplus BIO-2000 (São Paulo, Brazil) using commercial kits (Labtest, Lagoa Santa, Brazil) and following the suppliers’ recommended method.

### Statistical Analyses

Statistical analyses were performed using GraphPad Prism (ver. 6.00, La Jolla, California, USA). The normality of the data distribution was tested using the D’Agostino and Pearson, while the homoscedasticity of variance was evaluated with the Brown–Forsythe test. A log transformation [log­(*x*+1)] was applied for variables that did not follow a normal distribution. Differences between experimental groups were analyzed using one-way analysis of variance (ANOVA), followed by Tukey’s test for posthoc comparisons. *In vitro* studies assessing nuclear morphology were analyzed using two-way ANOVA, followed by the Bonferroni test. A *p*-value less than 0.05 (*p* < 0.05) was considered significant. Results are present as average ± standard deviation from at least three independent experiments.
